# From Uncertainty to Anxiety: How Uncertainty Fuels Anxiety in a Process Mediated by Intolerance of Uncertainty

**DOI:** 10.1155/2020/8866386

**Published:** 2020-11-22

**Authors:** Yuanyuan Gu, Simeng Gu, Yi Lei, Hong Li

**Affiliations:** Institute for Brain and Psychological Sciences, Sichuan Normal University, Chengdu 610066, China

## Abstract

Uncertainty about future events may lead to worry, anxiety, even inability to function. The highly related concept—intolerance of uncertainty (IU)—emerged in the early 1990s, which is further developed into a transdiagnostic risk factor in multiple forms of anxiety disorders. Interests in uncertainty and intolerance of uncertainty have rapidly increased in recent years; little is known about the construct and phenomenology of uncertainty and IU and the association between them. In an attempt to reveal the nature of two concepts, we reviewed broad literature surrounding uncertainty and intolerance of uncertainty (IU). We followed the process in which the whole IU theory developed and extended, including two aspects: (1) from uncertainty to intolerance of uncertainty and (2) definition of uncertainty and intolerance of uncertainty, and further concluded uncertainty fuels to negative emotions, biased expectancy, and inflexible response. Secondly, this paper summarized the experimental research concerning uncertainty and IU, consisted of three parts: (1) uncertainty-based research, (2) measurements of IU, and (3) domain-specific IU. Lastly, we pointed out what remains unknown and needed to be investigated in future research. This result provides a comprehensive overview in this domain, enhancing our understanding of uncertainty and IU and contributing to further theoretical and empirical explorations.

## 1. Introduction

We live in a world filled with uncertainty. Weather forecast often reports that there is an 80% chance of rain; the possibility never reaches 100%. We cannot be sure about what is going to happen next. What important is how it affects us and how we live and cope with it. Numerous researches have investigated the power of uncertainty, although some of them implicitly include it but did not claim for it. The dictionary definition of uncertainty is “experiencing an unknown” and closely related to unpredictability, ambiguity, unfamiliarity, etc. It is a tremendously hard work to review all the research concerning uncertainty and come to a common conclusion due to the broad definition and confusion between similar concepts. Additionally, some of them are often used interchangeably under certain circumstances ([1]). Here, we narrow down the field by arbitrarily defining the concept of uncertainty which only refers to different contingencies between environmental events in this review [2]. This leads to a clear cut between uncertainty and other similar concepts, such as ambiguity which results from features perceived as equivocal or perceived with insufficient knowledge for a singular definitive interpretation (e.g., figure/ground images like the Rubin vase; apophenia) [3]. Many research use unpredictability to describe stimulus contingencies, whose connotation is consistent with uncertainty according to our definition, allowing us to be as inclusive as possible and regard those two concepts as the same in terms of uncertainty studies. Consequently, studies that are regarding the specific aspect of uncertainty will be subjected to review no matter what particular word they use.

There is an increasing research interest in intolerance of uncertainty and its role in emotional disorders, which broadly refers to response to uncertainty in cognitive, emotional, and behavioral levels [4]. Existing literatures surrounding IU confirm that IU is a key construct in anxiety and worry, but little is known about its exact nature [5]. This paper traces IU definition back to the fundamental component which is uncertainty, attempting to clarify our understanding of both uncertainty and IU. In line with this thought, there are multiple dimensions of uncertainty, for example, (1) whether an environmental event will occur at all, (2) what kind of environmental event will occur, negative, neutral, or positive, and (3) when will an environmental event occur. So this paper mainly focuses on future-orientated uncertainty and IU construct, hoping to bring insight into uncertainty-related domain.

## 2. Theoretical Foundation of IU

### 2.1. From Uncertainty to IU

Many studies have historically demonstrated that uncertainty as a common feature in threat context may elicit fear and anxiety. The idea of “intolerance of uncertainty” emerges from anxiety-related studies; the key component of which was originally identified as fear of the unknown. Psychologists defined fear of the unknown as “an individual's propensity to experience fear caused by the perceived absence of information at any level of consciousness or point of processing” [6]. This fundamental fear is the basic cognitive process underlying all anxiety disorders [7], but different from emotional experience caused by anxiety. Fear is present-oriented and relatively certain while anxiety is future-oriented and relatively uncertain. Uncertainty is a central feature in the conceptual model of fear, which leads to anxiety and worry.

Decades of studies concerning uncertainty lay the foundation of intolerance of uncertainty construct. Unlike unpredictability and uncontrollability, uncertainty is a more diverse and inclusive concept which refers to any forms of unknown and can be specified in different research domains. Prior to the publication of IU construct, responses to uncertainty have been observed in fear, anxiety, and worry. Researchers observed that different emotional responses are affected by the degree of perceived uncertainty: fear is associated with less uncertain future threat while anxiety is related to more uncertain future threat [8]. Also affected by uncertainty are behaviors under environment with no explicit instructions. Intolerant response is a general description and identical feature of these phenomenological experiences in uncertainty-based studies which further conceptualized as IU construct.

### 2.2. Definition of Intolerance of Uncertainty

In 1994, Freeston et al. defined IU as “cognitive, emotional, and behavioral reactions to uncertainty in everyday life situations.” Based on this, researchers revised the definition of IU to clarify its nature, such as adding perception process, and features of uncertain situations [9]. These early definitions sought to include the overall influence of uncertainty, but recent trends are more focused on the cognitive level. Carleton et al. [10] emphasized that individuals high in IU would find possible future negative events unacceptable and threatening regardless of the probability of its occurrence. There is a very similar concept existing prior to IU referred to as “intolerance of ambiguity” [11]. It is necessary to differentiate those two construct, but the distinction between them was very obscure until Krohne [12] proposed that ambiguity of a situation is determined by its unpredictability, complexity, and insolubility. And he further suggested that ambiguity leads to uncertainty, which implies the consistency between them. The definition of intolerance of ambiguity also semantic overlaps with IU which refers to the tendency to perceive ambiguous situations as sources of threat [13].

It is premature to conclude that intolerance of uncertainty is an independent factor underlying high order construct due to the broad and inconsistent definition. The definition, at its core, is about the negative influence brought by uncertainty, such as fear, anxiety, and behavioral inhibition. Previous research shows that intolerance of uncertainty is highly associated with worry in clinical and nonclinical populations. According to the comprehensive definition of worry, it is rooted in thoughts that uncertain future events are negative accompanied by the feeling of anxiety [14]. Definitions of IU and worry demonstrate the fact that worry and IU share many common features regarding future uncertainty and uncertainty-induced maladaptive responses. Noticing that pathological worry is the hallmark of generalized anxiety disorder, it is not surprising that IU can distinguish participants with generalized anxiety disorder (GAD) from healthy controls. Researchers investigated the specificity of the relationship between IU and worry and found that IU was highly related to worry than to obsessions/compulsion and panic sensations ([15]). Does this close association reflect the nature of IU or just confusion in definitions? The definition of IU, at its core, is about the negative influence brought by uncertainty, such as fear, anxiety, and behavioral inhibition. To clear this confusion, researchers in this domain need to extend the conceptual construct from the originator of IU rather than empirical observations.

## 3. Experimental Research

### 3.1. Uncertainty-Based Research

Uncertainty regarding future events is inherently implicated in anxiety and worry due to its impact on our emotional state. It has been proposed that anxiety originates from excessive fear overgeneralization which “is quite likely that the summed frequency and intensity of the fear responses of any given individual to clear and imminent physical or psychological threat … would lag far behind the summed amount of fear in response to the anticipation of such events and the myriad anxious “what if …” mental representations of possible future events that are common in daily life” [8]. As pointed out, possible future events may induce the abuse of the associate learning. There is uncertainty in the fear learning and generalizing process all the way through, but the role uncertainty plays in this process is complicated. Reinforcement rate, which has been thoroughly investigated in reinforcement learning studies, can be identified as one of the uncertainty characteristics that we discussed here: whether reinforcement will occur at all. Uncertain condition in which reinforcement rate is between 100% and 0 has different reaction patterns [16].

Uncertainty about a future event may disrupt the anticipatory process which is the key component of adaptive cognitive responses, leading to overestimation of the threat possibility and severity. Former research used fear conditioning paradigm to investigate expectancy for unconditioned stimulus in an unpredictable context and found that unpredictability induced contextual fear and chronic expectation of potential threats. Participants showed elevated US expectancy in unpredictable context compared to predictable context and sustained expectancy when unpredictable shock is over [17]. Grupe and Nitschke [18] also found evidence to further support this conclusion by using NPU test paradigm which consisted of certain aversive picture, certain neutral picture, and uncertain picture. Self-report data revealed biased expectancies of aversion following uncertain cues, suggesting that participants tend to expect negative events while tolerating uncertain cues. These findings can be interpreted into uncertainty-induced expectancy bias and disrupted anticipatory process. But this association is only observed between threat-related cues and aversive outcomes, neither between threat-irrelevant cues and aversive outcomes nor threat-related cues and neutral outcomes [19–21]. Anticipation or expectation of future events is a very important notion in the domain development and sustenance of anxiety. From an evolutionary and adaptive perspective, it is beneficial for individuals to show hypervigilance to uncertainty and always prepare for the negative outcome.

In addition, expectancy bias as a cognitive factor is also influenced by other coexisting cognitive factors, such as increased selective attention, disrupted sensory processing, and inadequate emotion regulation. The idea that unpredictable future events need more selective attention to make accurate predictions and search for strategies seems to make intuitive sense. A recent study confirmed that uncertainty appeared to enhance the attention allocation in both early and late cognitive processes. Specifically, defensive response concerning uncertainty and prediction errors elicited different attentional dynamics in which participants received unexpected stimulus [22]. In line with this notion, there are other covariation biases regarding uncertainty; that is, uncertainty-related anxiety disrupts sensory processing and impairs the ability to assess stimulus contingencies. For instance, the so-called “illusory correlation” is describing that individual subjectively associate cues indicating potential threat with subsequent outcomes under unrelated circumstances ([18, 23]). It should be pointed out that these factors mentioned above are highly associated and effect cognitive process in different processing stages. Future studies need to clarify the cognitive mechanism of uncertainty processing and disentangle these factors.

Previous researches have showed that uncertainty may affect the acquisition and evaluation process of the predictive properties of uncertain environmental event. Lin et al. [24] conducted a ERP study using classic experimental paradigm called S1-S2 paradigm in which S1 (e.g., a question mark “?” stands for uncertain valence) are cues about certain or uncertain emotional valence of an upcoming event (S2) (e.g., a positive picture) ([25–27]). Results implicated that uncertain cues elicited larger N2 than the certain cues did about both future positive and negative pictures and produced smaller early contingent negative variation (CNV) than did the certain cues about future negative pictures. The results suggest that different attention and anticipation process occurs at the early cognitive processing stages of uncertainty ([24, 28]). What is more, Dieterich et al. [22] used similar paradigm and found that uncertain cues elicited increased P2 and LPP compared to certain cues. It is conceivable that predictable and unpredictable cues have different brain response pattern and uncertainty appears to increase the engagement of early phasic and sustained attention for uncertain events. Taken together, this finding was consistent with previous studies proving uncertainty is associated with biased expectancies and heightened response to aversive stimulus [18, 29, 30]; it also successfully detected uncertainty's modulation on response to positive events. Former studies using similar paradigm failed to find or have sufficient statistical power to detect the analogous effect of uncertainty on response to neutral and positive events ([18]).

Meanwhile, there is also evidence from NPU-threat test which is a developed form of S1-S2 paradigm and consists of three conditions: (1) N condition: no threat; (2) P condition: predictable threat; (3) U condition: unpredictable threat, demonstrating that the modulation effect of uncertainty may be specific to negative events [21]. The most studied predictability in NPU-threat test is temporal and valence predictability which is making the future event onset time and valence predictable. Parisi et al., [31] found that the impact of temporal uncertainty on startle magnitude is not evident for positive or neutral pictures and did not vary as a function of the emotional valence. One plausible explanation for the divergence is that temporary uncertainty and valence uncertainty may be inherently different and have different modulation patterns. Moreover, an individual may have different psychological magnitudes of impending positive and negative events. Notably, the effect of valence uncertainty appears to be consistent giving thought that participants' response to negative stimulus is generally more evident than to neutral and positive stimulus in experiments using NPU paradigm. That leads to one assumption which is uncertainty is specific to aversive events, even uncertainty itself is considered aversive or negative. Further explanation is that negative events might be more arousing and capture more attention relative to positive events such as scary pictures. In addition, an individual is more sensitive to negative events after controlling the effect of arousal level.

Another perspective is that uncertainty impairs our ability to prepare for and response to future events, thus contributing to anxiety, worry, and even fear ([1]). Regardless what kind of external event will occur, the uncertain waiting process before it finally onset contributes to the feeling of distress and worry. Uncertainty is threatening and dangerous, so we have to avoid them and be sure about something likely to happen. Even though we do not know what will happen or whether the worst results that we assume may or may not happen, we just find it hard to tolerate uncertainty and those negative feelings brought by it ([18]). Existing literatures already confirm that faster response can be achieved in a less uncertain condition. Take timing tasks as an example which includes temporal uncertainty; behavioral results showed that reaction time was significantly shorter in cued condition in which a cue signaled the exact onset time of stimulus and allowed participant to make temporal orientation than in an uncued condition where no cue was provided even though the accuracy was controlled. In addition, accuracy in both conditions was very high; both cued and uncued conditions were very high and showed no difference; one possible explanation was the ceiling effect [32]. At the same time evaluating the elapsed time to anticipate the onset time of forthcoming event, researchers use different approaches to decrease uncertainty during this period, such as providing certain cues to inform participants the exact onset time of stimulus. Certain cues can be divided into implicit (e.g., temporal template) and explicit cues (sensory cues which signal the onset time of stimulus) [33]; both implicit and explicit cues help participants to decrease the perceived uncertainty. Results indicate that decreasing uncertainty can speed up reaction; in another word, increasing uncertainty leads to behavioral inhibition demonstrated by disrupting and slowing down the planned action.

### 3.2. Measurements of IU

The 27-item Intolerance of Uncertainty Scale is the very first scale to measure IU which is developed on the basis of clinical observations [4]. It is empirical and largely based on descriptive statements which are extending the understanding of the broad definition by using a 5-point Likert scale ranging from 1 to 5. Despite this, IUS showed a highly consisted validity in psychometric testing. It has excellent internal consistency (0.91) and good discriminant and convergent validity and shows good performance as a criterion to distinguish participants with nonclinical GAD from healthy controls [4]. Although the theoretical foundation of IUS is not very solid and we do not know what it exactly measures, it has a face valid construct and has been replicated by latter researchers. Studies use IUS as a standard measurement to obtain the score of IU and distinguish high and low IU groups in their experiments [34, 35] or to investigate the association between IU and multiple emotion disorders and many other factors [36–39].

A large body of research conducts factor analysis of IUS in order to clarify the nature of this construct due to the confounding statement terms which seem similar to terms in trait and state anxiety scale. Birrell et al. [5] carried out an inclusive review regarding factor analyses of IUS and found that there may be two factors that are consistent throughout exploratory factor analyses. These two factors were consistently found to group together across different studies and samples. Therefore, it is necessary to test this two-factor structure of IU using different forms of scale and establish a clear and stable construct for future research. In addition, Carleton et al., [10] tested the fit of unitary-, four-, and five-factor model for 27-item IUS, and results showed that none of these models demonstrated adequate fit. The researchers also found a high-level redundancy within the scale due to the semantic overlap between items. Although the number of factors remains inconsistent in confirmatory factor analyses, a two-factor structure is preferred and supported by many studies which can be described as “unacceptability and avoidance of uncertainty” and “uncertainty leading to the inability to act” [4, 40]. Recent evidence suggests 2 factors can be interpreted into prospective factor (P-IU) and inhibitory factor (I-IU), and P-IU and I-IU are found to be associated with excessive and inflexible avoidance behavior [41] and processing uncertain errors [42]. While P-IU is identical to anxiety disorders characterized by expectancy bias [43] and increased emotional response, I-IU is specifically related to panic disorder and social anxiety [36].

Great efforts have been made to propose different versions of the original 27-item IUS to promote the understanding and application of IU construct. The short form of IUS has 12 items and identically high internal consistencies with two-factor model with less redundancy. It also has excellent convergent validity with the original and been tested repeatedly in clinical and nonclinical samples [44]. The modified version for children gains increased research interest which is adapted from the adult version. Different from adult IU, child IU may be related to more internalizing problems ([45]). Cornacchio et al. [46] performed a study on IUS for children to date its factor construct and found that IUSC shared 2-factor model with IUS-12. But it is too soon to conclude that the prospective/inhibitory model is the general construct underlying all items due to the inconsistent findings in factor analysis studies, and there may be a more ideal construct to conceptualize IU definition.

### 3.3. Domain-Specific IU

**Figure 1:**
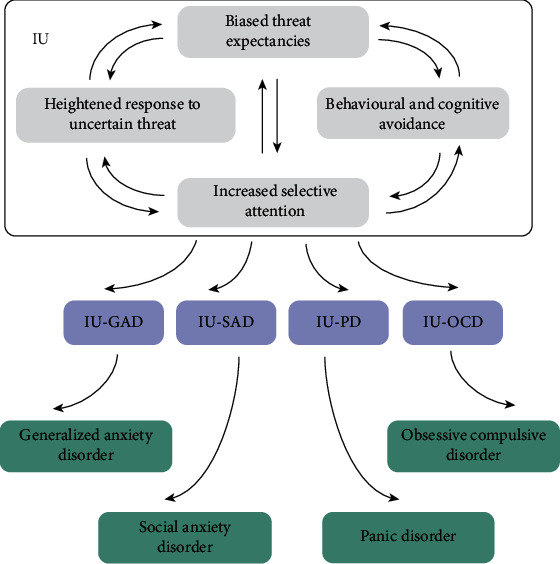
This figure demonstrates the cognitive model of IU across anxiety disorders raised by us. The IU construct consists of multiple factors and the interaction between them. In a process shown in the figure, different combinations.

Considerable amount of research have provided evidence that IU is a transdiagnostic dispositional risk factor for the development and maintenance of clinically significant anxiety [3]. Increasing findings demonstrate that IU is associated with a broad range of emotional disorders and other cognitive vulnerability factors, highlighting the theoretical and therapeutic importance of IU [47–49]. Recent researchers argue that disorder-specific IU is more strongly related to disorder symptoms than general IU trait [50]. Dugas et al. investigated the relationship between IU and worry in a nonclinical sample and found that IU was highly related to worry, moderately related to obsessions/compulsions, and weakly related to panic sensations. This pattern revealed that IU is specific to worry since IU was more highly correlated with worry relative to obsessions/compulsions and panic sensation ([15]). Several studies adopted different questionnaires and analytical method to examine the specificity of IU to particular psychological disorders in clinical and nonclinical samples. Evidence was found for the strong association between IU and GAD or OCD symptoms. However, researchers argued that IU got specific association with OCD demonstrated by the strongest association between IU and OCD than that between worry and GAD. Further, individuals with analogue GAD or OCD reported more intolerance of uncertainty than controls, but they did not differ significantly from each other ([47, 51]). To enhance the knowledge on the generality and specificity of IU, Paul et al. also conducted regression analyses among IU and symptom levels of GAD, social anxiety, OCD, and depression. They found that IU explained a significant amount of variance in social anxiety severity when controlling for established cognitive correlates of social anxiety (e.g., fear of negative evaluation) and for neuroticism. In addition, IU appeared to be associated with symptom levels of GAD, OCD, and social anxiety, but not depression, when controlling the share variance among these symptoms [52]. Taken together, we illustrated the complex association of IU and the process from IU to anxiety disorders (shown in Figure 1).

Dugas et al.'s model of GAD illustrates that IU features in the cognitive process where GAD patients receive the mood state and life event inputs and reaction to uncertainty under everyday life situation. There are other cognitive factors in this model coexisting and associated with IU, such as “poor problem orientation” and “cognitive avoidance.” The question of how exactly are individuals with GAD influenced by these factors needs more clarification due to complicated mechanism. Recent studies use a hierarchical model to evaluate the unique contribution of IU in anxiety-related pathology. Direct and indirect effects between IU and symptoms are observed through disorder-specific and multiple cognitive vulnerabilities. This model suggests that IU may be a more primary predictor of multiple disorder symptoms than the next-level cognitive factors [53, 54]. In clinical perspective and for potential treatment purpose, IU can be divided into different disorder-specific ingredients which account for most of the difference between relative emotional disorders. Consist of results of factor analysis, it also can be represented as having two dimensions since it mainly causes two kinds of response—prospective response and inhibitory response known as prospective IU (i.e., the cognitively focused dimension of IU; e.g., unforeseen events upset me greatly) and inhibitory IU (i.e., the behaviorally focused dimension of IU; e.g., the smallest doubt can stop me from acting). Likewise, IU is an underlying feature of several emotional disorders, and different dimensions comprise the respective factors which account for different anxiety disorder symptoms [43].

## 4. Directions for Future Research

Many years of studies yield substantial evidence for the effects of uncertainty and IU construct. The current paper was set up to review the rich body of research concerning this special issue and inspire future researchers. We have to point out that there is relative paucity and unsolved questions in some research domain. Firstly, according to current theories on emotion and motivation, anticipation of emotionally salient stimuli impacts motivational states regardless of valence. It can be proposed that uncertainty during anticipation of upcoming positive events also affects motivation states and response. Uncertainty's duration is rather short which is between cue onset time and the outcome onset time. Once outcome occurs, the mystery is resolved and uncertainty no longer exists. Uncertainty may directly or indirectly mediate the relationship between anticipation and motivation states, and future experimental researches are needed to clarify uncertainty's multiple effects. It remains controversial whether uncertainty serves a role as “fire alarm” which biased the emotional experience towards negative regardless of the actual valence of future events [55]. Or on the contrary, uncertainty could heighten response to negative events while dampening response to positive events.

Secondly, increasing laboratorial studies shed light on temporal uncertainty-related design which generally used cues to provide temporal information for participants. There are mainly two different task types: exogenous-based and endogenous-based tasks. The former relies on the external cues which have temporal predictive properties to inform the occurrence of stimulus, whereas the latter relies on participants' temporal conception to estimate the stimulus onset time due to the lack of explicit signal. Furthermore, there seems to be a paradox in experiments which manipulate uncertainty by providing an extra signal since signal itself is the opposite of uncertainty. For comparison, signal in control condition does not provide predictive information or does not occur while it does in experimental condition. In fact, invalid signal still alerts participants to uncertainty.

Thirdly, none of the theoretical models of IU construct shows good fit factor analysis research. There are complex associations between cognitive vulnerabilities and overlapping transdiagnostic factors. But the specific process and underlying mechanism are still in a black box. Does IU construct interact with and predict other constructs? More models should be tested by future researchers to explore the determinate factors in IU construct. Last but not least, it is still unclear what role IU plays in the treatment of anxiety disorder, whether trait IU changes or disorder-specific IU changes after the therapy. Researchers need to investigate the mechanisms of such changes across different treatment interventions [56]. Due to the fact that IU is a cognitive bias, exposure treatment may not be sufficient to reduce trait IU. Removing an individual's quest for certainty and safety is not realistic. Changing the negative beliefs about uncertainty and improving coping strategies seem more practical. Generally speaking, clinical applications need to be continuously explored based on cognitive vulnerabilities in the anxiety disorder region.

## Data Availability

The original data are available upon request.
